# Transoral endoscopic thyroidectomy vestibular approach (TOETVA): pioneers's point of view

**DOI:** 10.20945/2359-3997000000424

**Published:** 2021-11-24

**Authors:** Antonio Augusto Tupinambá Bertelli, Renan Bezerra Lira, Antonio José Gonçalves, Jonathon Owen Russell, Ralph Patrick Tufano, Gianlorenzo Dionigi, Hoon Yub Kim, Angkoon Anuwong, Luiz Paulo Kowalski

**Affiliations:** 1 Faculdade de Ciências Médicas da Santa Casa de São Paulo São Paulo SP Brasil Faculdade de Ciências Médicas da Santa Casa de São Paulo, São Paulo, SP, Brasil; 2 A.C. Camargo Cancer Center São Paulo SP Brasil A.C. Camargo Cancer Center, São Paulo, SP, Brasil; 3 Universidade Johns Hopkins Estados Unidos da América América Universidade Johns Hopkins, Estados Unidos da América; 4 Universidade de Messina Itália Universidade de Messina, Itália.; 5 Korea University College of Medicine Coreia do Sul Korea University College of Medicine, Coreia do Sul; 6 Police General Hospital Bangkok Tailândia Police General Hospital Bangkok, Tailândia; 7 Universidade de São Paulo A.C. Camargo Cancer Center São Paulo SP Brasil Universidade de São Paulo, São Paulo, SP, Brasil; A.C. Camargo Cancer Center, São Paulo, SP, Brasil

DEAR EDITOR,

We read with interest the recent report by Tincani and cols. (1) commenting on the growing adoption of remote access thyroidectomy in Brazil, as well as the accompanying editorial by Dr Shaha (2). Both of these manuscripts were listed as opinions of “experts” in the field, but it is apparent that the “experts” did not have all available data at the time of their writing.

For example, the informed reader will be aware that there are extensive publications on “time spent on the operation (longer than open surgery) (3), need for conversion to the classic route (about 1%) (4), presence of bleeding (not clinically different), rate of infection (similar to open surgery), complications such as injury to the mental nerves (less than 1%) (5), and aesthetic complications such as anterior cervical skin trauma (less than 1%)”. Furthermore, concerns regarding the two dimensions or limits of the operative field with endoscopic visualization are not related to higher complications with recurrent laryngeal nerves or parathyroid glands, and parrot the concerns of general surgeons prior to adopting laparoscopy or sinus surgeons before performing endoscopic surgery, both of which are now considered standard of care.

The authors go on to discuss the ethics of moving forward with this procedure outside of a research protocol (determined not to be necessary by surgical ethicists) (6), a hypothetical risk of increased infections (not reported to be greater than open surgery), novel complications, esthetics and the financial burden. Each of these have been studied to date, and there are multiple publications on each of those topics clearly defining the issues. Besides that, we are more likely to measure and record every outcome variable with TOETVA unlike open surgery where there may not be the same attention to details especially of the cosmetic considerations (skin burn, rating of scar, etc.) (6). Finally, the author of the editorial suggests that the learning curve and complication rate may actually be greater than that reported if some cases are not included. To insinuate this is what's occurring in these studies is baseless and undermining. It misrepresents the significant data and evaluation of it that includes all initial cases in multiple series.

During the last two decades, various remote access thyroidectomy techniques have been developed to improve cosmetic results compared to open surgery (7). While many have criticized the role of these techniques given that a cervical incision heals well for many patients, it is also true that the cervical incision remains the most common postoperative adverse event, with approximately 80% of patients expressing concerns about their scar at some point (8). Other authors have demonstrated that nearly 10% of patients consider corrective plastic surgery nearly 5 years after surgery (9). In light of these findings, the search for a cosmetically superior approach continues to attract patients and surgeons who remain committed to achieving the best overall outcome.

As surgeons strive to listen to their patients, avoid paternalism and improve outcomes, progress will come fitfully. The slow progress of remote access thyroidectomy to date demonstrates the challenges inherent. Given that approximately 50% of patients are candidates to avoid a cervical incision and may desire to do so (10), it behooves surgeons to critically determine which techniques should be adopted and which should be discarded. These decisions should rely on data only, not opinion or any other non-scientific motivations. The data in the published literature unequivocally and unimpeachably show that in select patients in select centers with select dedicated surgeons, TOETVA is not experimental and is also an acceptable alternative to transcervical thyroidectomy.
